# External influences over Senegalese health financing policy: delaying universal health coverage?

**DOI:** 10.1093/heapol/czad108

**Published:** 2023-11-09

**Authors:** Valéry Ridde, Jean-Hugues Caffin, Fatoumata Hane

**Affiliations:** Université Paris Cité, IRD, Inserm, Ceped, 45, Rue des Saints Pères, Paris F-75006, France; Institut de Santé et Développement, Université Cheikh Anta Diop, Dakar, Senegal; CIRAD (Agricultural Research for Development), UMR ASTRE, Dakar, Senegal; IRL 3189 Environnement, Santé et Société UCAD, Université de Assane Seck de Ziguinchor, Ziguinchor, Sénégal

**Keywords:** Universal health coverage, policy instrument, Senegal, Paris Declaration, ownership

## Abstract

Senegal has long sought solutions to achieve universal health coverage (UHC). However, in a context dependent on international aid, the country faces multiple external pressures to choose policy instruments. In this commentary, we propose an analysis of this influence. The empirical material comes from our involvement in analysing health reforms for 20 years and from many interviews and observations. While studies have shown that community-based health insurance (CBHI) was not an appropriate solution for UHC, some international actors have influenced their continued application. Another global partner proposed an alternative (professional and departmental CBHI), which was counteracted and delayed. These issues of powers and influences of international and national consultants, established in a neo-liberal approach to health, have lost at least a decade from UHC in Senegal. The alternative now appears to be acquired and is scaling up at the country level, witnessing a change in the current policy paradigm.

Key messages

- The choice of policy instruments for universal health coverage (UHC) is often not neutral
- Experts should provide conclusive and understandable evidence on the policy instruments they propose
- Policymakers in countries dependent on aid should be given the means to understand the rationale, relevance and adaptation of the instruments presented by financial partners
- Financial partners need to better align with the Paris Declaration and strengthen their coordination in proposed policy instruments

Senegal has long researched optimal solutions for implementing universal health coverage (UHC). In the early 2000s, multiple technical and financial partners (TFP) supported and financed community-based health insurance (CBHI) without coordination (Atim *et al.*, 2005; Alenda-Demoutiez, 2017). The UHC’s national policy (2010) and strategic development plan (2013–17) were based on these considerations, with a target of 75% of the population enrolled (Daff *et al.*, 2020; Paul *et al.*, 2020). The current meagre affiliation rate to communal CBHI (less than 5%) confirms that this policy instrument based on voluntary enrolment at low scale of risk pooling was not the most relevant (Waelkens *et al.*, 2017) even if the Belgian technical cooperation (Enabel project hereafter) proposed an alternative on a departmental scale (also based on voluntary enrolment) and with professional management (Bossyns *et al.*, 2018). Ten years later, the latter model has just gone to the national level. The commentary aims to show how specific TFPs have influenced the choice of policy instruments to the detriment of UHC in the case of CBHI.

## The adoption of the national program of communal CBHI

In 2013, the adoption of CBHI at the communal level (C-CBHI) as a national strategy (DECAM) followed the promotion of this model by U.S. Agency for International Development (USAID), which provided initial funding. As of 2010, the USAID Health Program encouraged the creation of the Inception and Policy Team (EIP), a working group led by the Cabinet of the Ministry of Health and Social Action (MSAS) to lead the reform. This influence was made possible due to the direct collaboration between the Cabinet General Secretary (leading the EIP) and the USAID Health programme manager, a retired civil servant of the MSAS who was also an official advisor to the influential General Director of Health. The EIP was asked to reflect on USAID’s three ‘proposed’ priorities: C-CBHI, performance-based financing and health emergencies. C-CBHI and the performance based financing (PBF) are the instruments of a global reform of health systems based on market logic (World Bank, 2003). They were the subject of national experimentation in Rwanda—involving Senegal experts—funded by USAID and World Bank (WB) (Paul *et al.*, 2018). C-CBHI is an autonomous micro-insurance; in a neo-liberal approach, this model (1) gives responsibility for funding to local actors and not the State, (2) opposes the principle of compulsory insurance managed by the State and (3) limits the risk at the lowest institutional level. World Health Organization does not recommend reliance on C-CBHI for UHC (Mathauer *et al.*, 2017).

USAID’s technical support, including a private consulting firm (Abt Associates), affected the choice and funding of the schemes. Abt was the structure responsible for the reform of health systems component and made significant contributions to roll out user fees in Africa (Lee and Goodman, 2002). Abt accompanied the realization of a mission of Senegalese decision-makers in Rwanda (2009), mainly composed of the members of the EIP. The Head of Abt Associates’ activities in Senegal—one of the key players in implementing C-CBHI in Rwanda between 1999 and 2004—facilitated this task. The mission, funded by the Bill and Melinda Gates Foundation and the Ministerial Leadership Initiative (MLI), aimed to support government practices (Caffin, 2018). In 2013, Abt supported the move of C-CBHI to a national level with the adoption of DECAM. The USAID proposed and adopted approaches without evidence, except for Rwanda’s success story (not really voluntary membership), and did not consider the public health policy debate and diverging interests (Rajkotia, 2018). The new MSAS research and statistical planning directorate (DRPS), created in 2013, could not assess the relevance of these policy instruments. This new direction was institutionally too weak to oppose any programme promoted by the Cabinet General Secretary and the General Director of Health. In addition, the DRPS has been rivalled by the monitoring unit of the national health development program (PNDS), which reports directly to the Minister’s Cabinet. The DRPS only obtained legal status in 2020. Also, the Enabel project was designed after the visit of the EIP in Rwanda. Cabinet members were already engaged in the USAID option before this new option emerged.

## An opposition by certain TFP

Between 2010 and 2017, Enabel (ex-Belgian cooperation) accompanied MSAS in implementing a programme targeting (1) strengthening national health governance and (2) reforming the management of health systems organized by professional at the department level (Enabel, 2017; Bossyns *et al.*, 2018). The five regions’ supportive service demand and supply (PAODES) project had 17 million Euros. The healthcare provision’s technical capacity of health centres was strengthened on the supply side, and a single unit flat price was created. On the demand side, departmental health insurance (UDAM) is aimed at a higher level than the communal (C-CBHI) to set up a team of professionals and ensure that the department outweighs the broader risk. The approach contrasts the model proposed by USAID and WB, as it is based on MSAS’s governance capacity. It assumed a regulation that limited the drifts linked to the commercialization of care while encouraging a professional CBHI model coordinated by the State (Enabel, 2017; Bossyns *et al.*, 2018).

The parties involved in implementing C-CBHI at the General Directory of Health and USAID office opposed the programme (Caffin, 2018), publicly complaining that Enabel was trying to promote his own ‘Belgium model’. The parties involved were mainly the Secretary General of the cabinet that had headed the EIP, the Director General of Health (one of whose advisors was at the same time coordinator of USAID’s health programme) and the former members of the EIP, some of whom were involved in taking the CBHI to scale. The launch of the Enabel project came as a counter-model to the solutions promoted by USAID when the pilot phase of the latter ended with a positive presentation justifying their move to a national scale. Enabel was asked to revise the model by following the C-CBHI of DECAM, even though the Enabel program (UDAM) was part of a financing agreement signed by the government. Enabel objected to this modification, noting the limitations of C-CBHI (management by non-professional volunteers, pooling on too small a scale, lack of portability, etc.) (Enabel, 2017) and the specificity of the Rwanda context limits its ability to serve as a model (Chemouni, 2018; Ridde *et al.*, 2018). Enabel objected to national ownership by refusing to comply with the DECAM, which has been seen as of exogenous origin (Caffin, 2018). In 2014, the Minister agreed to keep Enabel’s programme to compare the two approaches. As a result, the programme started several years later (Enabel, 2017). While PAODES faced many challenges, several attacks will be re-run. For example, the National Coordinator of PAODES was cleared by USAID with higher compensation. He was taken on—at a higher salary and with the agreement of the Ministry’s general secretariat, in the middle of the start-up phase of the Enabel project—to coordinate the competing project on PBF. His replacement was an HIV physician specialist—far from the necessary expertise (Bossyns *et al.*, 2018).

The Japanese technical cooperation (JICA), which was in the planning phase of its new project, was pressured to support the implementation of the model promoted by USAID and WB (Caffin, 2018). In support of the implementation of C-CBHI, the JICA representative, an advisor to the Ministry, was also under pressure from MSAS. At the same time, he thought this model needed to align with the Japanese experience. For him, introducing mandatory universal health insurance in Japan during the 1960s was a structural step in strengthening national feelings (Caffin, 2018). Therefore, the model supported by C-CBHI opposed the Japanese model. Research funded by JICA showed the C-CBHI model’s limitations and the appropriateness of professionalization (Rouyard *et al.*, 2022).

## The future of the UHC

The Enabel-funded programme started when the influence strategy of USAID and WB had already been launched for several years at the highest level for MSAS, with much greater resources. Its implementation was, therefore, limited in terms of time and resources (Enabel, 2017). However, the current success and sustainability of UDAM (Ridde *et al.*, 2022) in the face of fragmentation (Mladovsky, 2020) and chaotic changes in C-CBHI and very low public membership (Ly *et al.*, 2022) led the government to review its strategy. National evaluation of the UHC in 2020 led to discussions on these options (CRES, 2020). The technical services of the Ministry of Community Development, Social Welfare and Solidarity (MDCEST) recommended the departmentalization and professionalization of C-CBHI. In 2021, Enabel returned to supporting the first two UDAM and Lux-Dev, scaling up to several other departments until 2023. In 2022, 38 of the 46 departments wrote to National Agency for Universal Health Coverage (ANACMU), created in 2015, stating that they were interested in its adoption. In September 2022, ANACMU announced: ‘the restructuring of CBHI, from 676 communal CBHI to 46 departmental units’. Therefore, the State finally favoured the creation of insurance on a departmental scale.

On the one hand, it realized the inefficiency and ineffectiveness of the C-CBHIs, which had been known for a long time but were made clear by the national evaluation process in 2020 (CRES, 2020). On the other hand, the move to departmental units is based on the global evidence of the need for pooling at a higher level than the communes for greater portability and, above all, for the professionalization of management (Mathauer *et al.*, 2017; Ridde *et al.*, 2018). However, the newly organized departmental units have not imposed a single flat-rate pricing system on the supply side, a significant difference from the UDAM option. Finally, both models are still based on voluntary enrolment, which could be better, but larger risk polling at the department level can be seen as a preliminary step towards UHC. The Belgian project had indicated at the time of its launch that it was a step towards UHC, in which the States must play a central role and membership must become compulsory, whereas this was not an option envisaged by the model proposed by USAID. However, Senegal is now assisting a fundamental shift in approaches to most of the TFP, although the change still needs to convince the National Union of C-CBHIs. In early 2023, it issued a statement complaining about the lack of consultation and the ‘forcing’ of the ANACMU to ‘dissolve’ the C-CBHIs. The national union is concerned about the place of communities in the governance and organization of this reform and the departmental units.

## Conclusion

Why was so much time lost since the proposal for departmentalization in 2014 as a pre-step towards UHC than C-CBHIs? This commentary shows the influence of TFP in the choice of policy instruments (here C-CBHI), but also that the alignment and coordination of TFP, set out in the Paris Declaration, is far from being the rule in practice despite the rhetoric (Gautier and Ridde, 2017; Mladovsky *et al.*, 2023). The issues at stake in the choice of policy instruments (Lavigne Delville and Schlimmer, 2020), as seen for the COVID-19 pandemic in Senegal (Ridde and Faye, 2022), have delayed the progress to the UHC by more than a decade. Another ‘global health nonsense’ case study? (Stein *et al.*, 2022). A policy paradigm shift (Hall, 1993) in international support policies is urgent (Shroff *et al.*, 2022). Burkina Faso showed that this was possible. The State was at the heart of decision-making and funding its ambitious and efficient free care policy (Ridde and Yaméogo, 2018). However, in 2022, a new USAID execution agency has recruited the same consultants from Abt Associates and WHO for Senegal to support ANACMU in developing a strategy to introduce a systematic enrolment to CBHI (Ridde *et al.*, 2023). Only time will tell whether the government can implement another paradigm shift by organizing a compulsory membership to health insurance, as set out in its national health financing strategy since 2017 (Ministère de la Santé et de l’Action Sociale, 2017).

## Reflexivity Statement

The three authors, two French men and one Senegalese woman, have lived and worked in Senegal for a very long time. They reflected together on the content of the article and on the proposed analysis. The analysis is part of a reflexive and interdisciplinary approach in which the three authors were able to participate in the whole process of reflection. To facilitate the discussions, the article was first written in French and then translated with the help of Heather Hickey, whom we thank. The work was carried out without any specific funding.
